# 6,7,15,16-Tetra­hydro-5,14-dibutyl­benzo[1,2-*c*:4,5-*c*′]diacridine

**DOI:** 10.1107/S1600536812025962

**Published:** 2012-06-13

**Authors:** Xin-hua Lu, Jin Hu

**Affiliations:** a Department of Applied Chemistry, Nanjing College of Chemical Technology, Geguan Road No. 265 Nanjing, Nanjing 210048, People’s Republic of China; b Department of Chemical Engineering, Nanjing College of Chemical Technology, Geguan Road No. 265 Nanjing, Nanjing 210048, People’s Republic of China

## Abstract

The unit cell of the title compound, C_36_H_36_N_2_, contains two independent mol­ecules which are located about inversion centers. In each molecule the quinoline rings are almost planar, with mean deviations of 0.0302 (1) and 0.0173 (1) Å. In the crystal, mol­ecules are linked by C—H⋯π inter­actions into a three-dimensional network.

## Related literature

For background to the applications of the title compound, an important organic synthesis inter­mediate, see: Kolosov *et al.* (2002 ▶); Antoniadis *et al.* (1994 ▶); Tonzola *et al.* (2003 ▶). For the synthesis of the title compound, see: Crivello & Lam 1976 ▶).
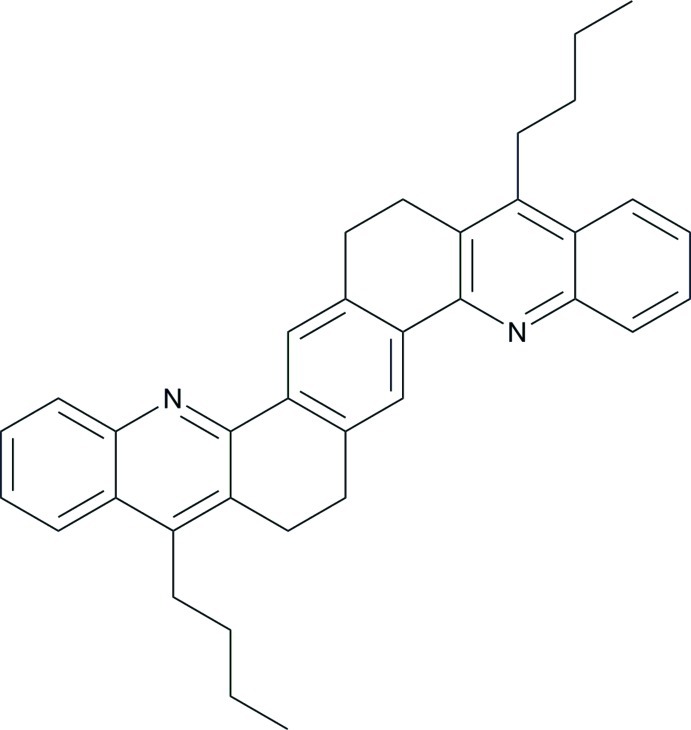
 Scheme faint, resolution poor

## Experimental

### 

#### Crystal data

C_36_H_36_N_2_
*M* *_r_* = 496.67
Triclinic, 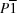

*a* = 9.6010 (19) Å
*b* = 10.386 (2) Å
*c* = 14.625 (3) Å
α = 77.44 (3)°
β = 78.43 (3)°
γ = 73.92 (3)°
*V* = 1352.4 (5) Å^3^
*Z* = 2
Mo *K*α radiation
μ = 0.07 mm^−1^
*T* = 293 K
0.30 × 0.20 × 0.10 mm

#### Data collection

Enraf–Nonius CAD-4 diffractometer
Absorption correction: ψ scan (North *et al.*, 1968 ▶) *T*_min_ = 0.979, *T*_max_ = 0.993
5301 measured reflections
4978 independent reflections
2825 reflections with *I* > 2σ(*I*)
*R*_int_ = 0.046
3 standard reflections every 200 reflections intensity decay: 1%

#### Refinement

*R*[*F* ^2^ > 2σ(*F* ^2^)] = 0.058
*wR*(*F* ^2^) = 0.147
*S* = 1.01
4978 reflections
344 parameters
H-atom parameters constrained
Δρ_max_ = 0.17 e Å^−3^
Δρ_min_ = −0.18 e Å^−3^

### 

Data collection: *CAD-4 Software* (Enraf–Nonius, 1985 ▶); cell refinement: *CAD-4 Software*; data reduction: *XCAD4* (Harms & Wocadlo, 1995 ▶); program(s) used to solve structure: *SHELXS97* (Sheldrick, 2008 ▶); program(s) used to refine structure: *SHELXL97* (Sheldrick, 2008 ▶); molecular graphics: *SHELXTL* (Sheldrick, 2008 ▶); software used to prepare material for publication: *SHELXTL*.

## Supplementary Material

Crystal structure: contains datablock(s) I, global. DOI: 10.1107/S1600536812025962/go2057sup1.cif

Structure factors: contains datablock(s) I. DOI: 10.1107/S1600536812025962/go2057Isup2.hkl

Additional supplementary materials:  crystallographic information; 3D view; checkCIF report

## Figures and Tables

**Table 1:** Hydrogen-bond geometry (Å, °) *Cg*23, *Cg*4 and *Cg*2 are the centroids of the of ring containing N2, the ring containing C15 and the ring containing C2, respectively.

*D*—H⋯*A*	*D*—H	H⋯*A*	*D*⋯*A*	*D*—H⋯*A*
C17—H17*B*⋯*Cg*23	0.97	2.79	3.688 (3)	155
C31—H31*A*⋯*Cg*4^i^	0.97	2.70	3.630 (3)	160
C34—H34*B*⋯*Cg*2^ii^	0.97	2.81	3.669 (3)	148

Symmetry codes: (i) 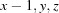; (ii) 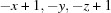.

**Fig. 1.**
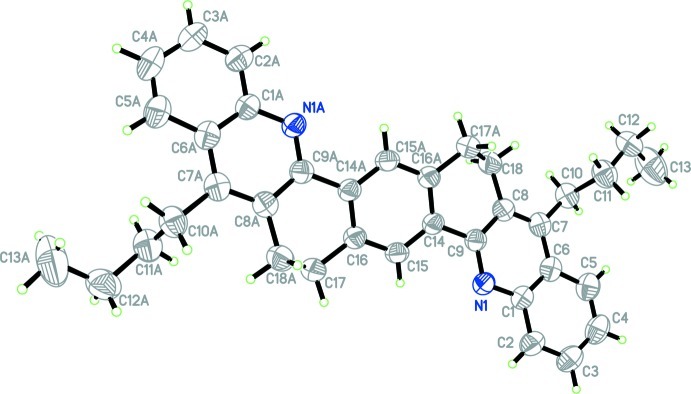
The molecular structure of (I), with the atom-numbering scheme. Displacement ellipsoids are drawn at the 50% probability level.

**Fig. 2.**
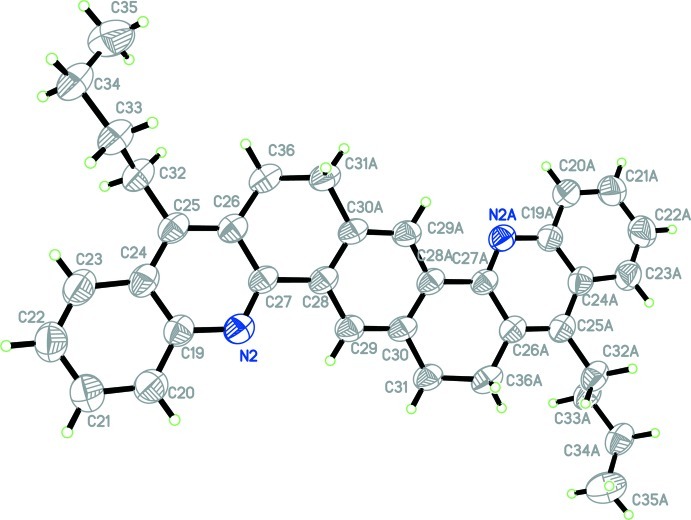
The second molecule in the asymmetric unit.

## supplementary crystallographic information

### Comment

The title compound is an new compound, which can be utilized to synthesize organic semiconductors and conjugated polymers (Tonzola *et al.*, 2003), which are of wide current interest for applications in electronic and optoelectronic devices including light-emitting diodes (Kolosov *et al.*, 2002), thin film transistors, and photovoltaic cells (Antoniadis *et al.*, 1994).

The molecular structure of (I) is shown in Fig. 1. The asymmetric unit contains two distinct title molecules of C_36_H_36_N_2_. In the molecules of C_36_H_36_N_2_, the quinoline rings are almost planar. The values of the mean deviation for quinoline rings are 0.0302 (1) Å and 0.0173 (1) Å respectively. The molecules are linked into a three-dimensional network by C—H···π interactions, C17–H17B···Cg23 links the two molecules in the asymmetric unit, C31–H31A···Cg4(-1+x,y,z) and C34–H34B···Cg2(1-x,-y,1-z), where Cg23, Cg4 and Cg2 contain atoms N2, C15 and C2 respectively.

### Experimental

The title compound, (I) was prepared by a method reported in literature (Crivello & Lam, 1976). The crystals were obtained by dissolving (I) (0.1 g) in methanol (30 ml) and evaporating the solvent slowly at room temperature for about 15 d.

### Refinement

All H atoms were positioned geometrically and constrained to ride on their parent atoms, with C—H(aromatic) = 0.93, Å C—H(CH_2_) 0.97Å with Uiso= 1.2Ueq(C) and C—H(methyl), 0.96Å with Uiso = 1.5Ueq(C).

### Crystal data

**Table:**

C_36_H_36_N_2_	*Z* = 2
*M**_r_* = 496.67	*F*(000) = 532
Triclinic, *P*1	*D*_x_ = 1.220 Mg m^−^^3^
Hall symbol: -P 1	Mo *K*α radiation, λ = 0.71073 Å
*a* = 9.6010 (19) Å	Cell parameters from 25 reflections
*b* = 10.386 (2) Å	θ = 9–13°
*c* = 14.625 (3) Å	µ = 0.07 mm^−^^1^
α = 77.44 (3)°	*T* = 293 K
β = 78.43 (3)°	Block, colourless
γ = 73.92 (3)°	0.30 × 0.20 × 0.10 mm
*V* = 1352.4 (5) Å^3^	

### Data collection

**Table:**

Enraf–Nonius CAD-4 diffractometer	2825 reflections with *I* > 2σ(*I*)
Radiation source: fine-focus sealed tube	*R*_int_ = 0.046
Graphite monochromator	θ_max_ = 25.4°, θ_min_ = 1.4°
ω/2θ scans	*h* = 0→11
Absorption correction: ψ scan (North *et al.*, 1968)	*k* = −12→12
*T*_min_ = 0.979, *T*_max_ = 0.993	*l* = −17→17
5301 measured reflections	3 standard reflections every 200 reflections
4978 independent reflections	intensity decay: 1%

### Refinement

**Table:**

Refinement on *F*^2^	Secondary atom site location: difference Fourier map
Least-squares matrix: full	Hydrogen site location: inferred from neighbouring sites
*R*[*F*^2^ > 2σ(*F*^2^)] = 0.058	H-atom parameters constrained
*wR*(*F*^2^) = 0.147	*w* = 1/[σ^2^(*F*_o_^2^) + (0.0519*P*)^2^ + 0.2931*P*] where *P* = (*F*_o_^2^ + 2*F*_c_^2^)/3
*S* = 1.01	(Δ/σ)_max_ < 0.001
4978 reflections	Δρ_max_ = 0.17 e Å^−^^3^
344 parameters	Δρ_min_ = −0.18 e Å^−^^3^
0 restraints	Extinction correction: *SHELXL*, Fc^*^=kFc[1+0.001xFc^2^λ^3^/sin(2θ)]^-1/4^
Primary atom site location: structure-invariant direct methods	Extinction coefficient: 0.0129 (14)

### Special details

**Table:**

Geometry. All e.s.d.'s (except the e.s.d. in the dihedral angle between two l.s. planes) are estimated using the full covariance matrix. The cell e.s.d.'s are taken into account individually in the estimation of e.s.d.'s in distances, angles and torsion angles; correlations between e.s.d.'s in cell parameters are only used when they are defined by crystal symmetry. An approximate (isotropic) treatment of cell e.s.d.'s is used for estimating e.s.d.'s involving l.s. planes.
Refinement. Refinement of *F*^2^ against ALL reflections. The weighted *R*-factor *wR* and goodness of fit *S* are based on *F*^2^, conventional *R*-factors *R* are based on *F*, with *F* set to zero for negative *F*^2^. The threshold expression of *F*^2^ > σ(*F*^2^) is used only for calculating *R*-factors(gt) *etc*. and is not relevant to the choice of reflections for refinement. *R*-factors based on *F*^2^ are statistically about twice as large as those based on *F*, and *R*- factors based on ALL data will be even larger.

### Fractional atomic coordinates and isotropic or equivalent isotropic displacement parameters (Å^2^)

**Table:**

	*x*	*y*	*z*	*U*_iso_*/*U*_eq_	
N1	0.2438 (2)	−0.0834 (2)	0.71474 (14)	0.0489 (5)	
C1	0.1692 (3)	−0.1557 (3)	0.78803 (17)	0.0484 (6)	
C2	0.0806 (3)	−0.0862 (3)	0.86002 (19)	0.0602 (8)	
H2	0.0721	0.0064	0.8549	0.072*	
C3	0.0073 (3)	−0.1526 (3)	0.9370 (2)	0.0677 (8)	
H3	−0.0506	−0.1055	0.9841	0.081*	
C4	0.0192 (3)	−0.2912 (3)	0.9450 (2)	0.0706 (9)	
H4	−0.0291	−0.3368	0.9984	0.085*	
C5	0.1004 (3)	−0.3604 (3)	0.8759 (2)	0.0643 (8)	
H5	0.1044	−0.4523	0.8818	0.077*	
C6	0.1794 (3)	−0.2955 (3)	0.79478 (18)	0.0501 (7)	
C7	0.2692 (3)	−0.3633 (3)	0.72059 (19)	0.0513 (7)	
C8	0.3490 (3)	−0.2911 (3)	0.64949 (18)	0.0492 (7)	
C9	0.3325 (3)	−0.1503 (3)	0.64924 (17)	0.0463 (6)	
C10	0.2797 (3)	−0.5116 (3)	0.7225 (2)	0.0587 (7)	
H10A	0.2959	−0.5280	0.6580	0.070*	
H10B	0.1873	−0.5323	0.7536	0.070*	
C11	0.4033 (3)	−0.6070 (3)	0.7741 (2)	0.0696 (9)	
H11A	0.4951	−0.5835	0.7451	0.084*	
H11B	0.3846	−0.5940	0.8396	0.084*	
C12	0.4181 (4)	−0.7567 (3)	0.7716 (2)	0.0849 (10)	
H12A	0.4283	−0.7682	0.7063	0.102*	
H12B	0.5064	−0.8111	0.7965	0.102*	
C13	0.2909 (4)	−0.8070 (4)	0.8270 (3)	0.1278 (16)	
H13A	0.3046	−0.9005	0.8221	0.192*	
H13B	0.2030	−0.7536	0.8027	0.192*	
H13C	0.2826	−0.7995	0.8923	0.192*	
C14	0.4177 (3)	−0.0722 (3)	0.57196 (16)	0.0453 (6)	
C15	0.3755 (3)	0.0684 (3)	0.55122 (17)	0.0478 (6)	
H15	0.2913	0.1144	0.5857	0.057*	
C16	0.4559 (3)	0.1419 (2)	0.48028 (17)	0.0470 (6)	
C17	0.4119 (3)	0.2944 (3)	0.45672 (19)	0.0560 (7)	
H17A	0.3495	0.3306	0.5108	0.067*	
H17B	0.3566	0.3216	0.4041	0.067*	
C18	0.4536 (3)	−0.3526 (3)	0.56941 (19)	0.0582 (7)	
H18A	0.4042	−0.3339	0.5145	0.070*	
H18B	0.4838	−0.4505	0.5884	0.070*	
N2	0.0362 (2)	0.3476 (2)	0.39887 (14)	0.0527 (6)	
C19	0.0721 (3)	0.4580 (3)	0.33770 (18)	0.0516 (7)	
C20	0.0175 (3)	0.5861 (3)	0.3663 (2)	0.0633 (8)	
H20	−0.0381	0.5922	0.4258	0.076*	
C21	0.0446 (3)	0.7013 (3)	0.3081 (2)	0.0721 (9)	
H21	0.0071	0.7853	0.3276	0.087*	
C22	0.1296 (3)	0.6918 (3)	0.2190 (2)	0.0778 (9)	
H22	0.1477	0.7702	0.1790	0.093*	
C23	0.1860 (3)	0.5692 (3)	0.1901 (2)	0.0698 (9)	
H23	0.2428	0.5653	0.1308	0.084*	
C24	0.1601 (3)	0.4472 (3)	0.24841 (18)	0.0540 (7)	
C25	0.2160 (3)	0.3153 (3)	0.22227 (17)	0.0514 (7)	
C26	0.1829 (3)	0.2053 (3)	0.28583 (17)	0.0484 (6)	
C27	0.0882 (3)	0.2267 (3)	0.37299 (17)	0.0462 (6)	
C28	0.0434 (3)	0.1101 (3)	0.43810 (17)	0.0474 (6)	
C29	−0.0784 (3)	0.1306 (3)	0.50862 (18)	0.0504 (7)	
H29	−0.1315	0.2189	0.5142	0.060*	
C30	−0.1223 (3)	0.0226 (3)	0.57047 (17)	0.0484 (7)	
C31	−0.2556 (3)	0.0434 (3)	0.64468 (18)	0.0549 (7)	
H31A	−0.3394	0.0372	0.6197	0.066*	
H31B	−0.2758	0.1338	0.6599	0.066*	
C32	0.3110 (3)	0.3004 (3)	0.12746 (17)	0.0576 (7)	
H32A	0.2735	0.3769	0.0802	0.069*	
H32B	0.3054	0.2181	0.1088	0.069*	
C33	0.4715 (3)	0.2940 (3)	0.12995 (18)	0.0614 (8)	
H33A	0.4761	0.3742	0.1519	0.074*	
H33B	0.5099	0.2150	0.1752	0.074*	
C34	0.5678 (3)	0.2860 (3)	0.03419 (19)	0.0687 (9)	
H34A	0.5232	0.3593	−0.0126	0.082*	
H34B	0.6624	0.2995	0.0380	0.082*	
C35	0.5909 (4)	0.1531 (4)	0.0016 (3)	0.1012 (12)	
H35A	0.6519	0.1544	−0.0591	0.152*	
H35B	0.4979	0.1398	−0.0036	0.152*	
H35C	0.6375	0.0802	0.0466	0.152*	
C36	0.2353 (3)	0.0614 (3)	0.26561 (18)	0.0593 (7)	
H36A	0.3278	0.0524	0.2231	0.071*	
H36B	0.1647	0.0436	0.2340	0.071*	

### Atomic displacement parameters (Å^2^)

**Table:**

	*U*^11^	*U*^22^	*U*^33^	*U*^12^	*U*^13^	*U*^23^
N1	0.0501 (13)	0.0521 (13)	0.0426 (12)	−0.0117 (11)	−0.0038 (11)	−0.0076 (10)
C1	0.0457 (15)	0.0557 (17)	0.0456 (15)	−0.0160 (13)	−0.0072 (13)	−0.0074 (13)
C2	0.0566 (18)	0.070 (2)	0.0517 (17)	−0.0150 (15)	−0.0016 (15)	−0.0124 (15)
C3	0.0606 (19)	0.086 (2)	0.0555 (19)	−0.0249 (17)	0.0058 (15)	−0.0136 (16)
C4	0.065 (2)	0.092 (3)	0.0564 (19)	−0.0374 (18)	0.0008 (16)	−0.0008 (17)
C5	0.0598 (18)	0.073 (2)	0.0630 (19)	−0.0289 (16)	−0.0078 (16)	−0.0025 (16)
C6	0.0418 (15)	0.0609 (18)	0.0508 (16)	−0.0178 (13)	−0.0134 (13)	−0.0032 (14)
C7	0.0474 (16)	0.0526 (17)	0.0568 (17)	−0.0140 (13)	−0.0164 (14)	−0.0049 (13)
C8	0.0522 (16)	0.0496 (17)	0.0484 (15)	−0.0128 (13)	−0.0114 (13)	−0.0097 (13)
C9	0.0475 (15)	0.0490 (16)	0.0416 (15)	−0.0081 (12)	−0.0097 (13)	−0.0079 (12)
C10	0.0562 (17)	0.0561 (18)	0.0684 (19)	−0.0200 (14)	−0.0146 (15)	−0.0077 (14)
C11	0.0596 (19)	0.065 (2)	0.082 (2)	−0.0155 (16)	−0.0184 (17)	−0.0023 (16)
C12	0.089 (3)	0.064 (2)	0.088 (2)	0.0050 (19)	−0.014 (2)	−0.0139 (18)
C13	0.112 (3)	0.079 (3)	0.188 (5)	−0.042 (2)	−0.032 (3)	0.019 (3)
C14	0.0476 (15)	0.0481 (16)	0.0382 (14)	−0.0074 (12)	−0.0065 (12)	−0.0082 (12)
C15	0.0446 (15)	0.0498 (17)	0.0446 (15)	−0.0033 (12)	−0.0035 (12)	−0.0124 (12)
C16	0.0498 (16)	0.0461 (16)	0.0424 (15)	−0.0069 (13)	−0.0067 (13)	−0.0085 (12)
C17	0.0567 (17)	0.0493 (17)	0.0553 (17)	−0.0038 (13)	−0.0042 (14)	−0.0108 (13)
C18	0.0668 (19)	0.0489 (17)	0.0585 (17)	−0.0105 (14)	−0.0090 (15)	−0.0132 (13)
N2	0.0486 (13)	0.0589 (15)	0.0485 (13)	−0.0124 (11)	−0.0039 (11)	−0.0091 (12)
C19	0.0442 (15)	0.0585 (18)	0.0492 (16)	−0.0112 (13)	−0.0075 (13)	−0.0046 (14)
C20	0.0628 (19)	0.067 (2)	0.0569 (18)	−0.0148 (16)	−0.0050 (15)	−0.0095 (16)
C21	0.075 (2)	0.062 (2)	0.075 (2)	−0.0171 (16)	−0.0064 (18)	−0.0061 (17)
C22	0.074 (2)	0.065 (2)	0.081 (2)	−0.0166 (18)	−0.0017 (19)	0.0054 (18)
C23	0.0600 (19)	0.077 (2)	0.0582 (19)	−0.0126 (17)	0.0006 (15)	0.0039 (17)
C24	0.0426 (15)	0.068 (2)	0.0486 (16)	−0.0159 (14)	−0.0062 (13)	−0.0008 (14)
C25	0.0377 (15)	0.070 (2)	0.0446 (15)	−0.0127 (13)	−0.0071 (12)	−0.0056 (14)
C26	0.0424 (15)	0.0599 (18)	0.0438 (15)	−0.0132 (13)	−0.0066 (12)	−0.0095 (13)
C27	0.0374 (14)	0.0602 (18)	0.0417 (15)	−0.0123 (13)	−0.0043 (12)	−0.0109 (13)
C28	0.0380 (14)	0.0615 (18)	0.0430 (15)	−0.0135 (13)	−0.0029 (12)	−0.0107 (13)
C29	0.0417 (15)	0.0557 (17)	0.0513 (16)	−0.0086 (12)	−0.0031 (13)	−0.0118 (13)
C30	0.0371 (14)	0.0607 (18)	0.0459 (15)	−0.0109 (13)	0.0005 (12)	−0.0139 (13)
C31	0.0429 (15)	0.0649 (18)	0.0529 (16)	−0.0107 (13)	0.0026 (13)	−0.0135 (14)
C32	0.0470 (16)	0.080 (2)	0.0444 (16)	−0.0184 (14)	−0.0050 (13)	−0.0061 (14)
C33	0.0485 (17)	0.084 (2)	0.0507 (17)	−0.0206 (15)	−0.0019 (14)	−0.0096 (15)
C34	0.0504 (17)	0.098 (3)	0.0561 (18)	−0.0258 (17)	0.0003 (15)	−0.0065 (17)
C35	0.088 (3)	0.117 (3)	0.094 (3)	−0.018 (2)	0.010 (2)	−0.042 (2)
C36	0.0523 (17)	0.078 (2)	0.0473 (16)	−0.0182 (15)	0.0034 (13)	−0.0170 (15)

### Geometric parameters (Å, º)

**Table:**

N1—C9	1.327 (3)	N2—C27	1.324 (3)
N1—C1	1.366 (3)	N2—C19	1.373 (3)
C1—C2	1.409 (3)	C19—C20	1.409 (4)
C1—C6	1.410 (3)	C19—C24	1.414 (3)
C2—C3	1.360 (4)	C20—C21	1.366 (4)
C2—H2	0.9300	C20—H20	0.9300
C3—C4	1.393 (4)	C21—C22	1.399 (4)
C3—H3	0.9300	C21—H21	0.9300
C4—C5	1.355 (4)	C22—C23	1.362 (4)
C4—H4	0.9300	C22—H22	0.9300
C5—C6	1.419 (4)	C23—C24	1.419 (4)
C5—H5	0.9300	C23—H23	0.9300
C6—C7	1.427 (4)	C24—C25	1.431 (4)
C7—C8	1.375 (3)	C25—C26	1.376 (3)
C7—C10	1.509 (3)	C25—C32	1.512 (3)
C8—C9	1.426 (3)	C26—C27	1.431 (3)
C8—C18	1.512 (3)	C26—C36	1.513 (4)
C9—C14	1.484 (3)	C27—C28	1.476 (3)
C10—C11	1.530 (4)	C28—C30^ii^	1.396 (3)
C10—H10A	0.9700	C28—C29	1.397 (3)
C10—H10B	0.9700	C29—C30	1.386 (3)
C11—C12	1.529 (4)	C29—H29	0.9300
C11—H11A	0.9700	C30—C28^ii^	1.396 (3)
C11—H11B	0.9700	C30—C31	1.502 (3)
C12—C13	1.481 (4)	C31—C36^ii^	1.519 (4)
C12—H12A	0.9700	C31—H31A	0.9700
C12—H12B	0.9700	C31—H31B	0.9700
C13—H13A	0.9600	C32—C33	1.531 (3)
C13—H13B	0.9600	C32—H32A	0.9700
C13—H13C	0.9600	C32—H32B	0.9700
C14—C15	1.384 (3)	C33—C34	1.521 (3)
C14—C16^i^	1.401 (3)	C33—H33A	0.9700
C15—C16	1.384 (3)	C33—H33B	0.9700
C15—H15	0.9300	C34—C35	1.502 (4)
C16—C14^i^	1.401 (3)	C34—H34A	0.9700
C16—C17	1.502 (3)	C34—H34B	0.9700
C17—C18^i^	1.520 (3)	C35—H35A	0.9600
C17—H17A	0.9700	C35—H35B	0.9600
C17—H17B	0.9700	C35—H35C	0.9600
C18—C17^i^	1.520 (3)	C36—C31^ii^	1.519 (4)
C18—H18A	0.9700	C36—H36A	0.9700
C18—H18B	0.9700	C36—H36B	0.9700
			
C9—N1—C1	117.5 (2)	C27—N2—C19	118.1 (2)
N1—C1—C2	117.6 (2)	N2—C19—C20	117.7 (2)
N1—C1—C6	122.9 (2)	N2—C19—C24	122.5 (2)
C2—C1—C6	119.5 (2)	C20—C19—C24	119.8 (3)
C3—C2—C1	120.9 (3)	C21—C20—C19	121.1 (3)
C3—C2—H2	119.5	C21—C20—H20	119.4
C1—C2—H2	119.5	C19—C20—H20	119.4
C2—C3—C4	119.8 (3)	C20—C21—C22	119.4 (3)
C2—C3—H3	120.1	C20—C21—H21	120.3
C4—C3—H3	120.1	C22—C21—H21	120.3
C5—C4—C3	120.7 (3)	C23—C22—C21	120.8 (3)
C5—C4—H4	119.6	C23—C22—H22	119.6
C3—C4—H4	119.6	C21—C22—H22	119.6
C4—C5—C6	121.3 (3)	C22—C23—C24	121.5 (3)
C4—C5—H5	119.4	C22—C23—H23	119.2
C6—C5—H5	119.4	C24—C23—H23	119.2
C1—C6—C5	117.6 (3)	C19—C24—C23	117.3 (3)
C1—C6—C7	118.4 (2)	C19—C24—C25	118.4 (2)
C5—C6—C7	124.0 (3)	C23—C24—C25	124.3 (3)
C8—C7—C6	118.2 (2)	C26—C25—C24	118.3 (2)
C8—C7—C10	121.2 (2)	C26—C25—C32	122.1 (3)
C6—C7—C10	120.6 (2)	C24—C25—C32	119.6 (2)
C7—C8—C9	119.2 (2)	C25—C26—C27	119.4 (2)
C7—C8—C18	123.5 (2)	C25—C26—C36	122.9 (2)
C9—C8—C18	117.3 (2)	C27—C26—C36	117.7 (2)
N1—C9—C8	123.6 (2)	N2—C27—C26	123.2 (2)
N1—C9—C14	117.2 (2)	N2—C27—C28	117.2 (2)
C8—C9—C14	119.2 (2)	C26—C27—C28	119.5 (2)
C7—C10—C11	112.8 (2)	C30^ii^—C28—C29	119.0 (2)
C7—C10—H10A	109.0	C30^ii^—C28—C27	120.2 (2)
C11—C10—H10A	109.0	C29—C28—C27	120.8 (2)
C7—C10—H10B	109.0	C30—C29—C28	121.7 (2)
C11—C10—H10B	109.0	C30—C29—H29	119.1
H10A—C10—H10B	107.8	C28—C29—H29	119.1
C12—C11—C10	112.5 (2)	C29—C30—C28^ii^	119.3 (2)
C12—C11—H11A	109.1	C29—C30—C31	122.1 (2)
C10—C11—H11A	109.1	C28^ii^—C30—C31	118.6 (2)
C12—C11—H11B	109.1	C30—C31—C36^ii^	111.8 (2)
C10—C11—H11B	109.1	C30—C31—H31A	109.3
H11A—C11—H11B	107.8	C36^ii^—C31—H31A	109.3
C13—C12—C11	112.8 (3)	C30—C31—H31B	109.3
C13—C12—H12A	109.0	C36^ii^—C31—H31B	109.3
C11—C12—H12A	109.0	H31A—C31—H31B	107.9
C13—C12—H12B	109.0	C25—C32—C33	112.3 (2)
C11—C12—H12B	109.0	C25—C32—H32A	109.1
H12A—C12—H12B	107.8	C33—C32—H32A	109.1
C12—C13—H13A	109.5	C25—C32—H32B	109.1
C12—C13—H13B	109.5	C33—C32—H32B	109.1
H13A—C13—H13B	109.5	H32A—C32—H32B	107.9
C12—C13—H13C	109.5	C34—C33—C32	113.3 (2)
H13A—C13—H13C	109.5	C34—C33—H33A	108.9
H13B—C13—H13C	109.5	C32—C33—H33A	108.9
C15—C14—C16^i^	119.4 (2)	C34—C33—H33B	108.9
C15—C14—C9	121.3 (2)	C32—C33—H33B	108.9
C16^i^—C14—C9	119.4 (2)	H33A—C33—H33B	107.7
C16—C15—C14	121.5 (2)	C35—C34—C33	113.4 (3)
C16—C15—H15	119.2	C35—C34—H34A	108.9
C14—C15—H15	119.2	C33—C34—H34A	108.9
C15—C16—C14^i^	119.1 (2)	C35—C34—H34B	108.9
C15—C16—C17	122.3 (2)	C33—C34—H34B	108.9
C14^i^—C16—C17	118.6 (2)	H34A—C34—H34B	107.7
C16—C17—C18^i^	110.7 (2)	C34—C35—H35A	109.5
C16—C17—H17A	109.5	C34—C35—H35B	109.5
C18^i^—C17—H17A	109.5	H35A—C35—H35B	109.5
C16—C17—H17B	109.5	C34—C35—H35C	109.5
C18^i^—C17—H17B	109.5	H35A—C35—H35C	109.5
H17A—C17—H17B	108.1	H35B—C35—H35C	109.5
C8—C18—C17^i^	110.8 (2)	C26—C36—C31^ii^	112.2 (2)
C8—C18—H18A	109.5	C26—C36—H36A	109.2
C17^i^—C18—H18A	109.5	C31^ii^—C36—H36A	109.2
C8—C18—H18B	109.5	C26—C36—H36B	109.2
C17^i^—C18—H18B	109.5	C31^ii^—C36—H36B	109.2
H18A—C18—H18B	108.1	H36A—C36—H36B	107.9
			
C9—N1—C1—C2	176.9 (2)	C27—N2—C19—C20	−179.4 (2)
C9—N1—C1—C6	−2.1 (3)	C27—N2—C19—C24	0.9 (4)
N1—C1—C2—C3	−177.6 (2)	N2—C19—C20—C21	−178.0 (3)
C6—C1—C2—C3	1.5 (4)	C24—C19—C20—C21	1.8 (4)
C1—C2—C3—C4	−0.2 (4)	C19—C20—C21—C22	−0.7 (4)
C2—C3—C4—C5	−1.6 (5)	C20—C21—C22—C23	−0.5 (5)
C3—C4—C5—C6	2.0 (4)	C21—C22—C23—C24	0.5 (5)
N1—C1—C6—C5	177.9 (2)	N2—C19—C24—C23	178.1 (2)
C2—C1—C6—C5	−1.0 (4)	C20—C19—C24—C23	−1.6 (4)
N1—C1—C6—C7	−1.4 (4)	N2—C19—C24—C25	−1.4 (4)
C2—C1—C6—C7	179.6 (2)	C20—C19—C24—C25	178.9 (2)
C4—C5—C6—C1	−0.7 (4)	C22—C23—C24—C19	0.5 (4)
C4—C5—C6—C7	178.6 (3)	C22—C23—C24—C25	179.9 (3)
C1—C6—C7—C8	4.3 (3)	C19—C24—C25—C26	−0.8 (4)
C5—C6—C7—C8	−175.0 (2)	C23—C24—C25—C26	179.7 (2)
C1—C6—C7—C10	−177.8 (2)	C19—C24—C25—C32	−179.7 (2)
C5—C6—C7—C10	2.9 (4)	C23—C24—C25—C32	0.9 (4)
C6—C7—C8—C9	−3.8 (4)	C24—C25—C26—C27	3.3 (3)
C10—C7—C8—C9	178.3 (2)	C32—C25—C26—C27	−177.8 (2)
C6—C7—C8—C18	177.0 (2)	C24—C25—C26—C36	179.7 (2)
C10—C7—C8—C18	−0.8 (4)	C32—C25—C26—C36	−1.5 (4)
C1—N1—C9—C8	2.7 (4)	C19—N2—C27—C26	1.8 (4)
C1—N1—C9—C14	−177.6 (2)	C19—N2—C27—C28	−177.6 (2)
C7—C8—C9—N1	0.3 (4)	C25—C26—C27—N2	−4.0 (4)
C18—C8—C9—N1	179.5 (2)	C36—C26—C27—N2	179.5 (2)
C7—C8—C9—C14	−179.4 (2)	C25—C26—C27—C28	175.4 (2)
C18—C8—C9—C14	−0.2 (3)	C36—C26—C27—C28	−1.2 (3)
C8—C7—C10—C11	88.0 (3)	N2—C27—C28—C30^ii^	−162.5 (2)
C6—C7—C10—C11	−89.8 (3)	C26—C27—C28—C30^ii^	18.1 (3)
C7—C10—C11—C12	−177.2 (3)	N2—C27—C28—C29	17.8 (3)
C10—C11—C12—C13	−67.4 (4)	C26—C27—C28—C29	−161.6 (2)
N1—C9—C14—C15	−19.7 (3)	C30^ii^—C28—C29—C30	0.4 (4)
C8—C9—C14—C15	160.1 (2)	C27—C28—C29—C30	−180.0 (2)
N1—C9—C14—C16^i^	159.6 (2)	C28—C29—C30—C28^ii^	−0.4 (4)
C8—C9—C14—C16^i^	−20.7 (3)	C28—C29—C30—C31	−178.2 (2)
C16^i^—C14—C15—C16	−0.3 (4)	C29—C30—C31—C36^ii^	−145.6 (2)
C9—C14—C15—C16	178.9 (2)	C28^ii^—C30—C31—C36^ii^	36.6 (3)
C14—C15—C16—C14^i^	0.3 (4)	C26—C25—C32—C33	−96.0 (3)
C14—C15—C16—C17	180.0 (2)	C24—C25—C32—C33	82.8 (3)
C15—C16—C17—C18^i^	143.2 (2)	C25—C32—C33—C34	−177.2 (2)
C14^i^—C16—C17—C18^i^	−37.2 (3)	C32—C33—C34—C35	−69.2 (3)
C7—C8—C18—C17^i^	−142.9 (3)	C25—C26—C36—C31^ii^	150.5 (2)
C9—C8—C18—C17^i^	38.0 (3)	C27—C26—C36—C31^ii^	−33.1 (3)

Symmetry codes: (i) −*x*+1, −*y*, −*z*+1; (ii) −*x*, −*y*, −*z*+1.

### Hydrogen-bond geometry (Å, º)

Cg23, Cg4 and Cg2 are the centroids of the of ring containing N2, the ring containing C15 and the ring containing C2, respectively.

**Table:**

*D*—H···*A*	*D*—H	H···*A*	*D*···*A*	*D*—H···*A*
C17—H17*B*···*Cg*23	0.97	2.79	3.688 (3)	155
C31—H31*A*···*Cg*4^iii^	0.97	2.70	3.630 (3)	160
C34—H34*B*···*Cg*2^i^	0.97	2.81	3.669 (3)	148

Symmetry codes: (i) −*x*+1, −*y*, −*z*+1; (iii) *x*−1, *y*, *z*.
